# Analysis of the Effects of Various Analgesia Methods on Post-cesarean Pain and Their Correlation with Inflammatory Biomarkers

**DOI:** 10.5812/aapm-166560

**Published:** 2026-01-04

**Authors:** Thaer Kareem Oleiwi Atabi, Ali Jabbari, Parvaneh Ebrahimzadeh, Hamzeh Bader Gazal, Somayeh Ghorbani Gholiabad, Ali Movafegh

**Affiliations:** 1 Department of Anesthesiology and Critical Care, International Faculty, Golestan University of Medical Sciences, Gorgan, Iran; 2 Department of Anesthesiology and Critical Care, Ischemic Disorders Research Center, Faculty of Medicine, Golestan University of Medical Sciences, Gorgan, Iran; 3 Department of Anesthesiology and Critical Care, Sayyad Shirazi Hospital, Golestan University of Medical Sciences, Gorgan, Iran; 4 Department of Anesthesia and Intensive Care, Al Zahra'a Teaching Hospital, Kut, Iraq; 5 Cancer Research Center, Golestan University of Medical Sciences, Gorgan, Iran; 6 Department of Anesthesiology, School of Medicine, Tehran University of Medical Sciences, Tehran, Iran

**Keywords:** Cesarean Section, General Anesthesia, Patient-Controlled Analgesia, Pain Measurement, Inflammation, Transversus Abdominis Plane Block

## Abstract

**Background:**

Post-cesarean section (CS) pain satisfaction remains an issue. The purpose of this research was to evaluate the effectiveness of the transversus abdominis plane (TAP) block in comparison to intravenous analgesia controlled by patients for managing pain after CS in Iraq.

**Objectives:**

The study aimed to evaluate pain intensity as the primary outcome, alongside secondary outcomes including vital signs, nausea, vomiting, medication use, and inflammatory markers.

**Methods:**

A quasi-experimental study was conducted at Wasit Investment Hospital in Kut, Iraq, involving 78 pregnant women undergoing elective CS. Sampling was conducted among eligible women who signed an informed consent form. Participants were classified into two groups based on the type of analgesia received after CS. The first group included women who received a TAP block using bupivacaine (n = 39). The second group consisted of those who used a patient-controlled analgesia (PCA) pump containing nalbuphine (n = 39). Pain intensity was measured using the Short Form McGill Pain Questionnaire (SF-MPQ; Arabic version) at 2, 4, 6, 12, and 24 hours following the CS. Laboratory tests, including a complete blood cell count (CBC) and high-sensitivity C-reactive protein (hs-CRP), were performed 24 hours after surgery.

**Results:**

There were no notable differences in the demographic, clinical, or laboratory characteristics between groups (P > 0.05). Pain levels assessed using the SF-MPQ at 2, 4, and 6 hours post-surgery were notably lower in the TAP block group than in the PCA group (P = 0.009, P = 0.005, and P = 0.001, respectively). A positive and significant relationship between hs-CRP levels and pain intensity was identified across all measurement times in the TAP block group.

**Conclusions:**

The findings of this study showed that the use of a TAP block technique provided more effective pain relief than PCA during the first 6 hours after a CS.

## 1. Background

Recent literature reports a significant increase in the worldwide cesarean delivery rate. This rise is linked to greater awareness among women and increased demand for pain-free techniques during and after surgery (1). Women who have a cesarean section (CS) typically experience significant pain in the initial hours following the surgery. For about one in ten of them, this pain may linger for up to six months after the operation. Inadequately managed pain after a CS can negatively impact the mother-infant bond by interfering with breastfeeding (2). Consequently, ensuring effective post-CS pain relief is an important issue for both mothers and obstetric anesthesiologists.

In recent years, the ultrasound-guided transversus abdominis plane (TAP) block has become increasingly recognized as an effective pain management technique for patients undergoing various abdominal surgeries (3). Most studies, primarily randomized clinical trials, have confirmed the effectiveness of the TAP block analgesia technique and have suggested it as the preferred method of analgesia for people who are contraindicated for the prescription of opioids or who are not qualified to undergo spinal anesthesia (4).

In a CS, a TAP block is a suitable option for pain relief in women who are prohibited from receiving neuraxial morphine for various reasons (5). This technique is used as an adjunctive palliation method that helps reduce opioid dependence during surgery as well as postoperative pain management (5). Transversus abdominis plane block analgesia is specifically applied to the internal oblique and transverse abdominal muscles within the fascial area, effectively blocking the thoracolumbar nerves in this region. The main branches of the nerves in this area extend between these two muscle groups and divide into the lateral and anterior cutaneous nerves near the midline of the axilla (5, 6).

Patient-controlled analgesia (PCA) decreases the need for injectable opioids while enhancing patient satisfaction through effective pain relief (7). Patient-controlled analgesia is especially recommended for women during labor or after CSs due to its programmable dosing and injection intervals (7). In this way, patients can self-administer a predetermined bolus dose of medication as needed. Each bolus can be given on its own or alongside other drugs (8). Patient-controlled analgesia is commonly used to treat chronic, acute, labor-related, and postoperative pain. The medications most often used are local anesthetics and opioids; however, other analgesics can also be employed (8, 9).

Women often experience dissatisfaction with pain management after CS. Effectively assessing the intensity of post-CS pain is crucial for choosing the proper analgesic method, medication, and dosage, which can improve pain relief after surgery. Our objective was to evaluate and compare two methods of analgesia, TAP block and intravenous PCA, for relieving pain after CSs in Iraq in 2024.

## 2. Methods

### 2.1. Study Design and Participant Inclusion Criteria

This study was conducted at Wasit Investment Hospital in Kut, Iraq, over six months from September 20, 2024, to March 30, 2025. The hospital's medical board granted ethical approval for this study after they reviewed the ethical guidelines (IR.GOUMS.REC.1403.010). In this quasi-experimental study, post-CS women who received two different methods of postoperative analgesia, including TAP block and PCA, were compared in terms of pain intensity within 24 hours after surgery. The inclusion criteria for the study were as follows: Participants had to be aged 40 years or younger, have a gestational age between 37 and 39 weeks, maintain a hemoglobin level ≥ 11 g/dL, have a singleton pregnancy, and undergo CS under general anesthesia. Pregnant women with the following medical conditions were excluded from the study: Coagulation disorders, diabetes, allergies to analgesic drugs, chronic pelvic pain, depression, or any psychological disorders. Consequently, all women included in the study were classified as ASA I according to the American Society of Anesthesiologists (ASA) classification. Samples were selected from all women who met the study criteria and signed the informed consent form.

The sample size was calculated based on the findings of Salem et al. (10) and using G*Power. The calculation was based on the difference in pain intensity means between two groups of women who underwent TAP block anesthesia and IV PCA anesthesia two hours after CS. After accounting for a 20% attrition rate, the final sample size was 78 participants, with 39 women per group (effect size: 0.830, an error: 0.05, b: 0.1).

The sample size was calculated based on the findings of Salem et al. (10) and using G*Power (version 3.1.9.4). The calculation was based on the mean difference of pain intensity two hours after CS between two groups of women who had received a TAP block or IV PCA for analgesia. After accounting for a 20% attrition rate, the final sample size was 78 participants, with 39 women per group (effect size: 0.830, α: 0.05, β: 0.1). All procedures performed during this study were part of the treatment process, and participants did not receive any additional interventions before, during, or after the CS.

### 2.2. Transversus Abdominis Plane Block and Patient-Controlled Analgesia Administration

General anesthesia was initiated with propofol (2.5 mg/kg), ketamine (1 mg/kg), and rocuronium (1 mg/kg), after which orotracheal intubation was performed. Mechanical ventilation was used, and anesthesia was sustained with isoflurane (at a concentration of 0.8% - 1.2%). Following delivery, anesthesia was maintained through IV injections of a combination of 2.5 mg midazolam, 0.3 µg/kg fentanyl, and 0.2 mg/kg etomidate.

Following the procedure and while adhering to aseptic techniques, the anesthesiologist employed ultrasound guidance to administer the TAP block through a single injection using a 25-gauge needle. The needle was carefully directed under the ultrasound probe until it penetrated the abdominal area. To prevent injections into the intraperitoneal, intramuscular, or intravascular spaces, the probe was aligned with the entry point of the needle. A total of 20 mL of 0.25% bupivacaine, mixed with normal saline, was injected into the fascial space situated between the internal oblique and transversus abdominis muscles on both sides. All TAP blocks were conducted by the same anesthesiologist.

In the PCA group, the proper use of the PCA pump had been thoroughly explained to patients in the recovery room after CS. The pump solution contained nalbuphine at a concentration of 0.5 mg/mL, with a volume of 100 cc. This pump administered a 1 mg bolus and had a lockout period of 10 minutes.

In both groups, if patients had experienced unbearable pain intensity and restlessness, 100 mg diclofenac suppositories had been prescribed.

### 2.3. Short Form McGill Pain Questionnaire

The Short-Form McGill Pain Questionnaire (SF-MPQ; Arabic version), developed and adapted by Terkawi et al. (11), was used to assess pain intensity at 2, 4, 6, 12, and 24 hours after CS. The questionnaire consists of 15 questions designed to measure pain intensity using a 4-point Likert scale (ranging from 0 to 3). It encompasses two main dimensions: Sensory (11 questions) and affective (4 questions). Additionally, the questionnaire includes an 11-point numeric rating score (NRS) (ranging from 0 to 10), a 6-point rated Present Pain Intensity (PPI) Index (from no pain to excruciating), and a 3-point rated pain description (brief, intermittent, and continuous).

### 2.4. Data Collection and Outcomes

Participants in both groups were assessed within 24 hours of CS and after being discharged from the recovery room to the ward. The SF-MPQ was completed with the assistance of the patients' companions at intervals of 2, 4, 6, 12, and 24 hours after the CS. Full explanations of the study's importance, as well as instructions on how to complete the questionnaire at the appointed times, were provided to the patients' companions. To minimize bias and any potential misunderstandings regarding the questionnaire, a contact number was made available to the companions for any questions or clarifications they might need.

The main outcome measured was pain intensity at 2, 4, 6, 12, and 24 hours following CS. The secondary outcomes included measurement of heart rate and respiratory rate, nausea and vomiting, total of diclofenac suppositories prescription, blood indices, such as complete blood cell count (CBC) and its components, as well as inflammatory indices, including high-sensitivity C-reactive protein (hs-CRP), which were performed 24 hours after CS.

### 2.5. Data Analysis

SPSS version 25 and GraphPad Prism 5.04 were used to perform all statistical analyses. An independent *t*-test was used for parametric data, and the Mann-Whitney U test for nonparametric data to compare the mean values of the quantitative variables between the two analgesic groups. Two-way analysis of variance was performed to assess changes in pain intensity over time.

## 3. Results

**Figure 1.**
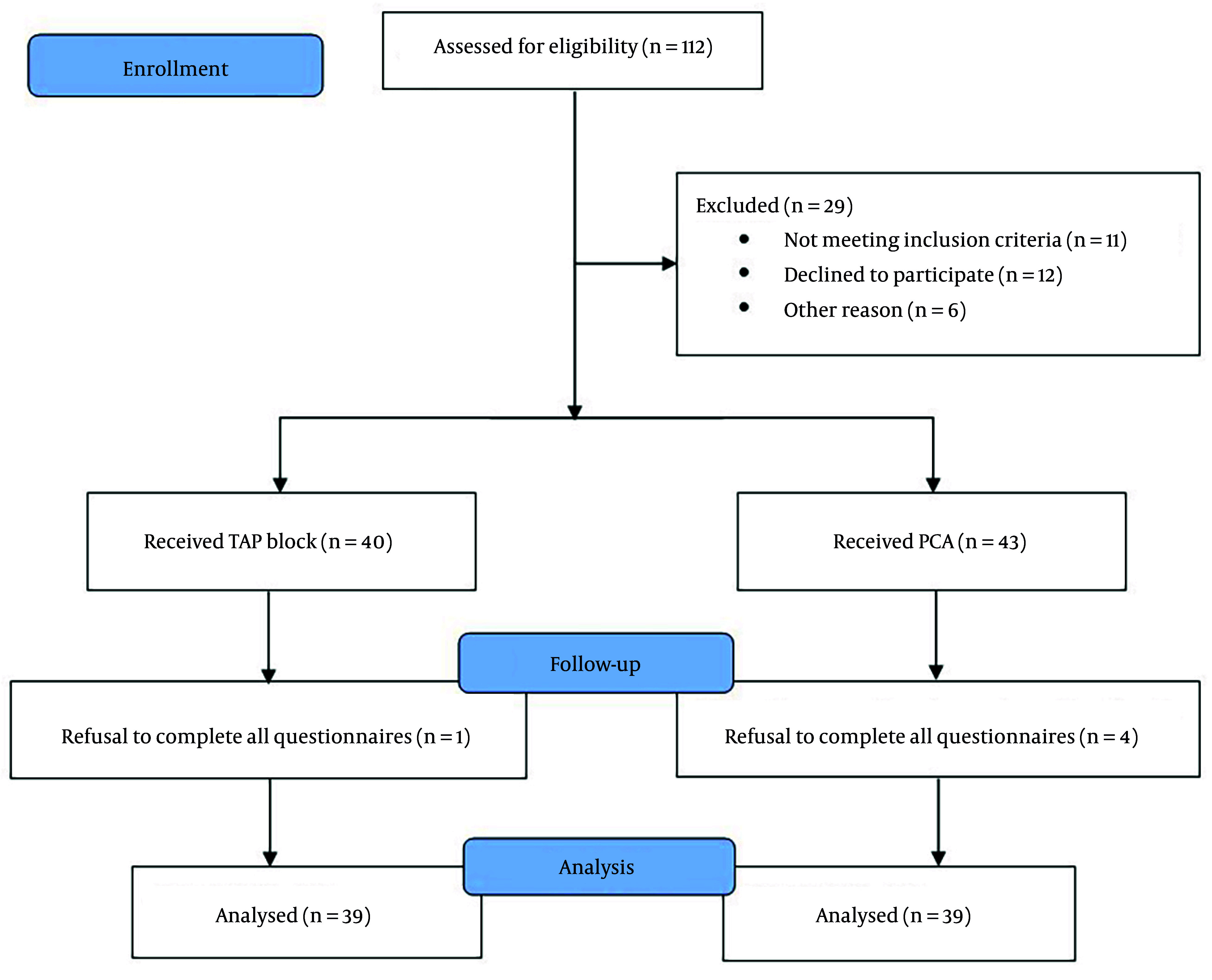
Flow diagram of study (abbreviations: PCA, patient-controlled analgesia; TAP, transversus abdominis plane)

Women who underwent CSs were divided into two groups based on the type of postoperative analgesia they received. After applying the exclusion criteria and after participants completed questionnaires, 39 women were included in each analgesia group (Figure 1). 

The participants' average ages were similar in both the TAP block and PCA groups, measuring 28.67 ± 5.67 and 29.79 ± 5.86 years, respectively (P = 0.212). Additionally, the duration of surgery was similar between the TAP block and PCA groups, measuring 72.05 ± 9.16 minutes and 72.44 ± 12.46 minutes, respectively (P = 0.561). The frequency of nausea and vomiting, and demand for diclofenac suppositories in the PCA group, was not significantly higher than in the TAP block group (P = 0.401 for nausea, P = 0.235 for vomiting, and P = 0.129 for diclofenac suppositories demand). There was no significant difference in heart and respiratory rates 24 hours after CS between the two groups (P > 0.05 for both). Other hematological and inflammatory indices were also comparable between the study groups (P > 0.05; Table 1). 

**Table 1.** Comparison of Clinical and Laboratory Characteristics Between Two Groups of Women Undergoing Cesarean Section with Patient-Controlled Analgesia and Transversus Abdominis Plane Block (n = 39) ^a, b^

Variables	TAP Block	PCA	P-Value
**Nausea ** ^**c**^	6 (15.4)	10 (25.6)	0.401
**Vomiting ** ^**c**^	2 (5.1)	5 (12.8)	0.235
**Heart rate ** ^**d**^	70.82 ± 3.28	72.08 ± 3.88	0.126
**Respiratory rate ** ^**e**^	16.28 ± 1.57	15.95 ± 1.47	0.251
**Diclofenac 100 mg ** ^**c**^	4 (10.3)	9 (23.1)	0.129
**Hemoglobin (g/dL) ** ^**e**^	12.67 ± 1.13	12.69 ± 1.16	0.869
**WBC (× 10^9^/L) ** ^**e**^	11.04 ± 3.74	10.48 ± 3.76	0.374
**Neutrophil (× 10^9^/L) ** ^**e**^	8.25± 3.56	7.86 ± 3.76	0.487
**Lymphocyte (× 10^9^/L) ** ^**e**^	2.22 ± 0.74	2.00 ± 0.67	0.192
**Platelet (× 10^9^/L) ** ^**d**^	222.69 ± 46.46	232.41 ± 74.03	0.490
**hs-CRP (ng/mL) ** ^**e**^	745.96 ± 264.44	815.88 ± 311.39	0.134

Abbreviations: TAP, transversus abdominis plane; PCA, patient-controlled analgesia; WBC, white blood cell; hs-CRP, high-sensitivity C-reactive protein.

^a^ Values are expressed as No. (%) or mean ± standard deviation.

^b^ There were no significant differences between the two groups, PCA and TAP block, in terms of demographic, clinical, and laboratory characteristics.

^c^ Chi-square test.

^d^ Independent *t*-test.

^e^ Mann-Whitney U-test.

**Figure 2.**
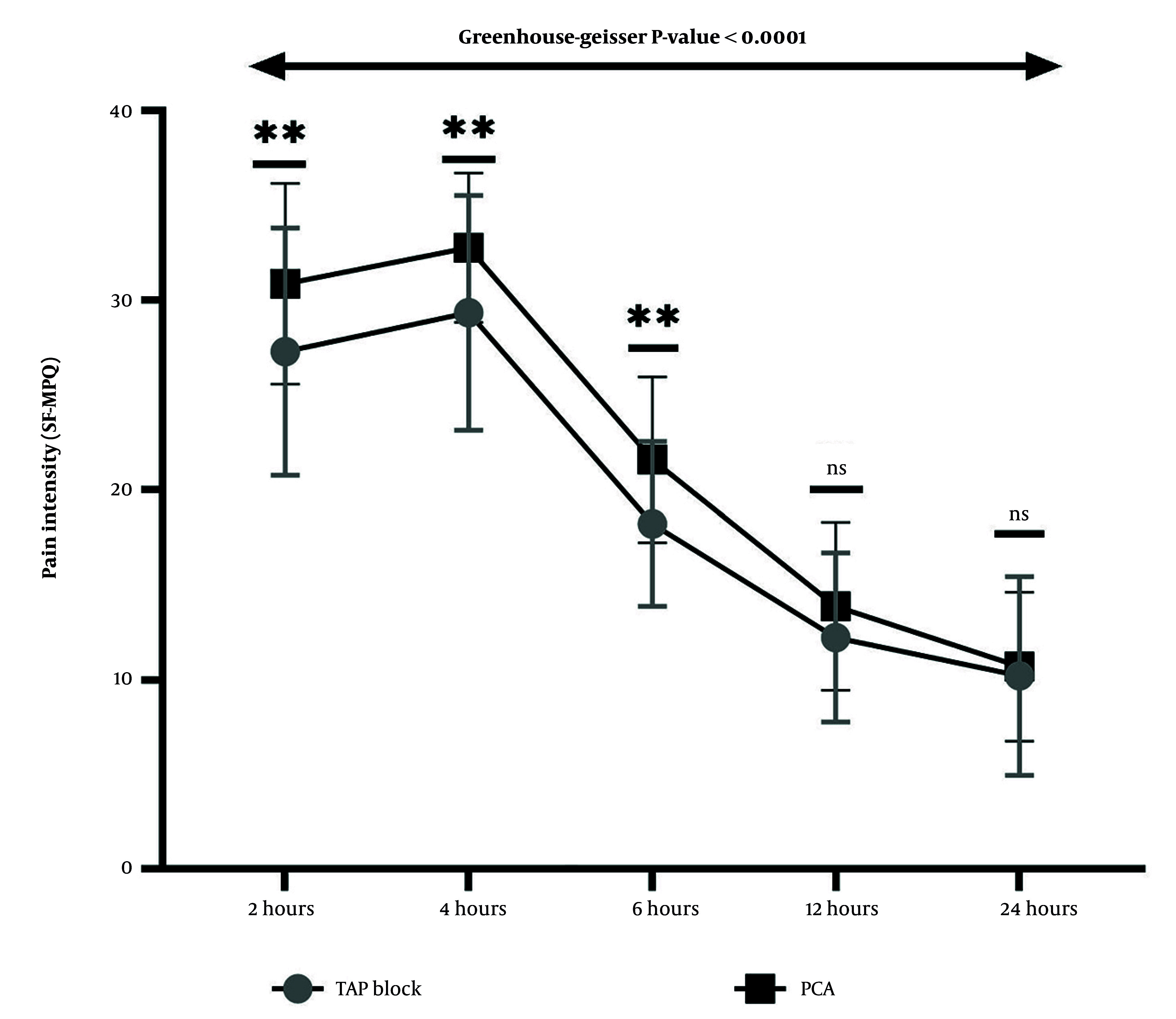
Trends and comparison of pain intensity based on Short Form McGill Pain Questionnaire (SF-MPQ) between two groups of patient-controlled analgesia (PCA) and transversus abdominis plane (TAP) block at 2, 4, 6, 12, and 24 hours after cesarean section (CS). At 2, 4, and 6 hours after CS, pain intensity was significantly higher in the PCA than in the TAP block group. All pairwise comparisons between the two analgesia groups were performed using independent *t*-tests (** P < 0.01). Repeated-measures ANOVA reveals that pain intensity at each measurement time in the two groups differs significantly from that at other times (Greenhouse-Geisser P-value < 0.0001).

Cronbach's alpha for the SF-MPQ was 0.871, which is considered good. As shown in Figure 2, pain intensity, as measured by the total SF-MPQ score, was significantly different between the two groups at 2, 4, and 6 hours after CS. At the first measurement of pain intensity, conducted 2 hours after CS, the TAP-block group experienced significantly lower pain levels than the PCA group, with a mean difference of -3.590 (95% Confidence Interval: -6.27 to -0.91, p-value = 0.009). Four hours after CS, pain intensity had increased in both groups compared to the first measurement. However, the TAP-block group still reported significantly lower pain levels than the PCA group, with a mean difference of -3.436 (95% Confidence Interval: -5.78 to -1.09, P-value = 0.005). Six hours after CS, pain intensity had decreased compared to the first and second measurements. Nonetheless, pain levels in the TAP-block group remained significantly lower than those in the PCA group, with a mean difference of -3.385 (95% Confidence Interval: -5.35 to -1.42, P-value = 0.001). Analysis of variance with repeated measures reveals that the pain intensity at each measurement time differs significantly from that at other times within each group (Greenhouse-Geisser < 0.0001). Pain intensity rose in the first 4 hours post-CS, then decreased until the 24 hours after surgery, the endpoint of measurements.

The comparison of pain intensity between the TAP block and PCA groups in the sensory dimension presented consistent results, as indicated by the total scores on the SF-MPQ. The pain intensity in the PCA group was notably greater compared to the TAP block at 2, 4, and 6 hours following the CS (P = 0.040, P = 0.005, and P = 0.003, respectively). In contrast, the comparison of pain intensity between the TAP block and PCA groups in the affective dimension showed some differences. The findings showed that the affective pain intensity in the PCA group was significantly higher than that in the TAP block at 2, 6, and 12 hours post-CS (P = 0.041, P = 0.001, and P < 0.001, respectively; Table 2). 

**Table 2.** Comparison of Pain Intensity in Two Sensory and Affective Dimensions of the Short Form of McGill Pain Questionnaire Between the Transversus Abdominis Plane Block and Patient-Controlled Analgesia Groups (n = 39) ^a, ^^b^

Variables	Sensory		P-Value	Affective		P-Value ^d^
	TAP Block	PCA		TAP Block	PCA	
**2 hours**	20.69 ± 5.73	23.54 ± 4.45	0.040 ^d^	6.59 ± 1.92	7.33 ± 1.71	0.041
**4 hours**	21.67 ± 5.79	24.85 ± 3.68	0.005 ^c^	7.67 ± 1.24	7.92 ± 0.90	0.487
**6 hours**	13.49 ± 3.50	15.85 ± 3.26	0.003 ^c^	4.72 ± 1.21	5.74 ± 1.53	0.001
**12 hours**	8.31 ± 3.71	8.64 ± 3.22	0.673 ^c^	3.92 ± 1.24	5.23 ± 1.65	< 0.001
**24 hours**	6.92 ± 4.02	7.74 ± 3.19	0.234 ^d^	3.28 ± 1.60	2.95 ± 1.36	0.218

Abbreviations: TAP, transversus abdominis plane; PCA, patient-controlled analgesia.

^a^ Values are expressed as mean ± standard deviation.

^b^ In the sensory dimension, pain intensity was significantly higher in the PCA group than in the TAP block group at 2, 4, and 6 hours after cesarean section. In the affective dimension, pain intensity was significantly higher in the PCA group than in the TAP block group at 2, 6, and 12 hours after cesarean section.

^c^ Independent *t*-test,

^d^ Mann-Whitney U-test.

**Figure 3.**
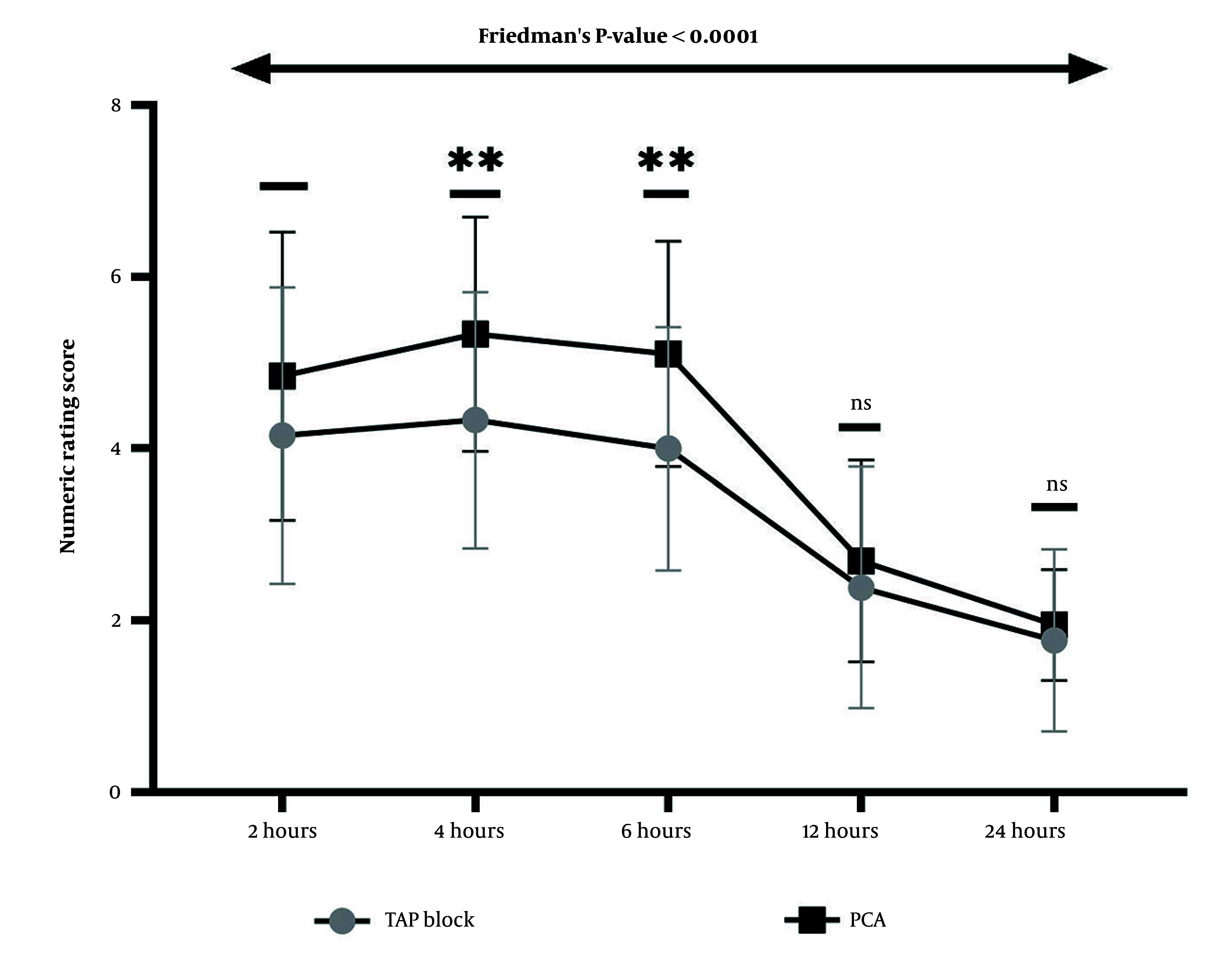
Trend and comparison of pain intensity based on the numeric rating score (NRS) between two groups: Patient-controlled analgesia (PCA) and transversus abdominis plane (TAP) block, at 2, 4, 6, 12, and 24 hours after cesarean section (CS). The Mann-Whitney U test showed that pain intensity was significantly higher at 4 and 6 hours after CS in the PCA group than in the TAP block group (** P < 0.01). In comparison, no significant difference was observed in pain intensity between the two analgesic methods at 2, 12, and 24 hours after surgery. Friedman's test reveals that the intensity of pain at each measurement time in both groups differs significantly from that at other times (Friedman's P-value < 0.0001 for both groups).

A comparison of pain intensity using the NRS between the two groups showed that at 4 and 6 hours after CS, pain intensity in the PCA group was significantly higher than in the TAP block group (P = 0.003 and P = 0.001, respectively). Friedman's two-way ANOVA revealed that pain intensity, as measured by the NRS, differed significantly in the two groups at each measurement compared to other times (Friedman's P-value < 0.0001; Figure 3). 

The results of the PPI Index showed that participants in the PCA group reported higher pain levels compared to those in the TAP block group at 2, 4, and 6 hours after CS, with p-values of 0.043, 0.018, and 0.009, respectively. However, the pain descriptions indicated no differences between the groups at any time point (p > 0.05; Table 3). 

**Table 3.** Comparison of Present Pain Intensity and Pain Description Indices Between Patient-Controlled Analgesia and Transversus Abdominis Plane Block Groups ^a^

Variables	Present Pain Intensity						P-Value	Pain Description			P-Value
	No Pain	Mild	Discomfort	Distressing	Horrible	Excruciating		Brief	Intermittent	Continues	
**2 hours**							0.043				0.422
TAP block	-	-	11 (28.2)	20 (51.3)	8 (20.5)	-		7 (17.9)	25 (64.1)	7 (17.9)	
PCA	-	-	3 (7.7)	22 (56.4)	14 (35.9)	-		4 (10.3)	24 (61.5)	11 (28.2)	
**4 hours**							0.018				0.070
TAP block	-	-	16 (41.0)	15 (38.5)	6 (15.5)	2 (5.1)		-	24 (61.5)	15 (38.5)	
PCA	-	-	4 (10.3)	21 (53.8)	9 (23.1)	5 (12.8)		-	16 (41.0)	23 (59.0)	
**6 hours**							0.009				0.150
TAP block	-	7 (17.9)	17 (43.6)	9 (23.1)	6 (15.4)	-		4 (10.3)	31 (79.4)	4 (10.3)	
PCA	-	1 (2.6)	9 (23.1)	15 (38.4)	14 (35.9)	-		1 (2.6)	29 (74.3)	9 (23.1)	
**12 hours**							0.551				0.131
TAP block	7 (17.9)	11 (28.2)	7 (17.9)	14 (35.9)	-	-		14 (35.9)	25 (64.1)	-	
PCA	6 (15.4)	17 (43.6)	6 (15.4)	10 (25.6)	-	-		8 (20.5)	31 (79.5)	-	
**24 hours**							0.582				0.648
TAP block	14 (35.9)	16 (41.0)	9 (23.1)	-	-	-		18 (46.2)	21 (53.8)	-	
PCA	13 (33.3)	20 (51.3)	6 (15.4)	-	-	-		16 (41)	23 (59)	-	

Abbreviations: PCA, patient-controlled analgesia; TAP, transversus abdominis plane.

^a^ Values are expressed as frequency (%).

^b^ The chi-square test is used for comparisons. Women undergoing cesarean sections who received PCA reported significantly different PPI levels at 2, 4, and 6 hours post-surgery compared to those who received a TAP block. No significant difference was observed in pain descriptions between the two groups at all the times examined.

Among all laboratory indicators measured, only hs-CRP showed a positive, significant correlation with pain intensity at all time points after CS in the TAP block group. No correlations were observed in the PCA group (Table 4). 

**Table 4.** Correlation Between Pain Intensity Based on Short Form McGill Pain Questionnaire and High-Sensitivity C-Reactive Protein at Measured Times After Cesarean Section in the Two Patient-Controlled Analgesia and Transversus Abdominis Plane Block Anesthesia Groups ^a^

Pain Intensity	hs-CRP				
	2 Hours	4 Hours	6 Hours	12 Hours	24 Hours
**TAP block**					
r ^b^	0.594	0.641	0.468	0.568	0.699
P-value	< 0.001	< 0.001	0.003	< 0.001	< 0.001
**PCA**					
r	0.089	-0.114	0.042	0.210	-0.228
P-value	0.589	0.489	0.797	0.200	0.163

Abbreviation: hs-CRP, high-sensitivity C-reactive protein; TAP, transversus abdominis plane; PCA, patient-controlled analgesia.

^a^ A positive and significant correlation was observed between hs-CRP levels and pain intensity at all measured times after cesarean section in the TAP block group. This correlation was not observed in the patient-controlled analgesia group.

^b^ Spearman correlation coefficient.

## 4. Discussion

This study was conducted to compare the effects of two analgesia methods on pain relief during the 24 hours after CS in an Iraqi population. The study's findings showed that the pain intensity of patients in both PCA and TAP block groups increased during the 4 hours after surgery and then gradually decreased over the next 20 hours. In this study, we demonstrated that patients who received TAP block tolerate less pain after CS than those under PCA. This difference in pain intensity was observed during the first 6 hours after CS and disappeared within 12 and 24 hours after the procedure.

The surgical TAP block is a new and easy technique for postoperative pain management that any obstetrician can learn. This effective method provides long-lasting analgesia and reduces the use of rescue analgesics. As an adjunct to multimodal pain management, the TAP block improves patient satisfaction after CS. Salem et al. indicated that intravenous PCA outperformed the TAP block because of its impact on the visceral response, whereas the TAP block was favored to prevent the systemic effects associated with the opioids used in PCA (10). The findings of the present study were in contrast to those of Salem et al. In the present study, pain intensity, as calculated by the SF-MPQ, was significantly lower during the first 6 hours after CS in the TAP block group compared to the PCA group. However, when pain intensity was measured using a numeric rating scale, this difference was observed only at 4 and 6 hours post-surgery. The observed difference between the two studies could be attributed to the use of different anesthesia techniques during the operation and the varying methods employed for recording postoperative pain intensity. Although the pain rating scale offers a one-dimensional view of pain, one of the strengths of this study is the use of the SF-MPQ. This tool assesses pain both qualitatively and quantitatively, allowing patients to provide more precise information to anesthesiologists. As a result, anesthesiologists can adjust postoperative analgesia by considering the different descriptions of pain intensity provided by patients (12, 13). Another study indicated that using a patient-controlled pethidine pump significantly reduced pain compared to a TAP block procedure with 0.25% bupivacaine (14).

The findings of analgesia in the present study were consistent with some other studies. One study found that using TAP block analgesia after laparotomies led to lower pain levels and reduced opioid requirements compared to a PCA method (15). Another study indicated that the use of TAP block analgesia after gynecological surgeries under general anesthesia significantly reduced the patients' pain 8 to 12 hours after surgery compared to the control group (16). In addition to the analgesia technique employed, one of the key reasons for the existence of conflicting evidence in analgesia studies is related to the type of drug and its concentration (17).

Understanding the type and severity of pain is crucial for selecting the most effective analgesia with minimal side effects, thereby reducing the patient's discomfort. The specific circumstances of the patient determine the choice between various opioid and non-opioid analgesic drugs (18). In the case of a CS, the mother's successful recovery with minimal pain is closely linked to the health of her baby. Therefore, it is essential to prescribe an effective analgesic that alleviates the postpartum pain while also minimizing side effects for both the mother and the baby (19).

Nalbuphine is a morphine-like analgesic that acts as an agonist on both μ and κ receptors, providing strong analgesic effects. This makes it a suitable option for alleviating gynecological pain, such as severe contractions during childbirth (20). The superior analgesic effects of nalbuphine, along with its low side effects and lack of neurotoxicity, have made it a suitable option for post-CS analgesia, which can be used alone or in combination with other analgesic agents, such as bupivacaine (20, 21). Bupivacaine is a long-acting local anesthetic that has been mainly used in operations under general anesthesia due to its good properties in controlling postoperative complications. The binding of this drug to plasma proteins has increased its half-life to approximately 5.5 hours (22). Numerous studies have examined various concentrations of this drug, both alone and in combination with other analgesic agents, to assess their effectiveness in reducing postoperative pain, often yielding conflicting results (23-25). In the present study, the TAP block and PCA groups were similar in terms of postoperative complications, including heart rate, respiratory rate, nausea, vomiting, and the need for diclofenac suppositories. This suggests that the combination of drugs used in both methods has a comparable effect in controlling post-CS complications.

The findings of our study exhibited that, although the level of the inflammatory marker hs-CRP was within the normal range in both analgesia groups, a significant and positive correlation was observed between hs-CRP levels 24 hours after CS and pain intensity at all measured times in the TAP block group. Postoperative pain is generally caused by inflammation. Bupivacaine can reduce pain intensity through various mechanisms, including epidural block, peripheral nerve block, subarachnoid block, and the blocking of sodium channels in the nerve membrane. Additionally, it increases the threshold of the action potential and decreases its speed, as well as the speed of nerve impulses (26, 27). The results of an in vivo study on a mouse model have demonstrated that bupivacaine reduces NF-κB expression and increases IκB expression, leading to effective relief of inflammation‑induced pain (28). A study has shown that in women undergoing CS with spinal anesthesia using 0.5% bupivacaine, the intensity of pain measured 6 hours after surgery showed a significant and positive correlation with hs-CRP levels before operation (29).

This study had several limitations. Since a randomized clinical trial was not feasible, we conducted a quasi-experimental study. Participants were selected from those available, and no randomization was used. Although informed consent was obtained from all participants before they entered the study, many expressed dissatisfaction with the presence of the study administrator at their bedside, prompting completion of questionnaires with the cooperation of the patients' companions. Although the study administrator trained these companions on how to administer the questions and when to complete the questionnaires, variations in how different subjects were asked could introduce bias. According to the companions, questionnaires were sometimes completed later than scheduled, particularly because many patients were sleepy or restless, especially during the first 6 hours after surgery. Nonetheless, at 12 and 24 hours postoperatively, the majority of participants completed the questionnaires themselves on schedule.

### 4.1. Conclusions

The findings of this study indicated that TAP block analgesia is more effective than PCA in relieving pain in women who have undergone CS within less than 12 hours after the procedure. However, postoperative outcomes, including nausea, vomiting, diclofenac usage, heart rate, respiratory rate, and hematological and inflammatory factors, did not show significant differences between the two analgesia methods. This study also revealed that the severity of pain, measured by the SF-MPQ, in women who had undergone CS and had received TAP block analgesia was directly correlated with the serum level of the inflammatory marker hs-CRP at all assessed time points.

## Data Availability

The dataset presented in the study is available on request from the corresponding author during submission or after publication.
